# Alendronate preserves femoral head shape and height/length ratios in an experimental rat model: A computer-assisted analysis

**DOI:** 10.4103/0019-5413.44630

**Published:** 2009

**Authors:** Eli Peled, Jacob Bejar, Chaim Zinman, Daniel N Reis, Jochanan H Boss, Hadar Ben-Noon, Doron Norman

**Affiliations:** Department of Orthopedic Surgery B, Rambam Health Care Campus, The Bruce Rappaport Faculty of Medicine, Technion-Israel Institute of Technology, Haifa, Israel; 1 Department of Pathology, B'nai Zion Medical Center, The Bruce Rappaport Faculty of Medicine, Technion-Israel Institute of Technology, Haifa, Israel

**Keywords:** Alendronate, avascular necrosis, femoral head, morphometry, rat model

## Abstract

**Background:**

Surgical osteonecrosis of the rat femoral head was induced by detaching the ligamentum teres and stripping the femoral neck periosteum. Bone and marrow necrosis were found from the fifth postoperative day and replaced by creeping substitution. Osteonecrosis of the femoral head results in the flattening to various degrees of roundness and osteoarthritic changes of the hip joint. Alendronate, an osteoclast inhibitor, slows down bone resorption and remodeling. The purpose of this study was to evaluate objectively the influence of alendronate treatment on the rat femoral head shape after six weeks of daily treatment, when compared with controls.

**Materials and Methods:**

The blood circulation of right femoral head of 20 female Sprague-Dawley rats was interrupted. Twelve were treated by alendronate injections of 200 µg/kg/day and eight controls were treated with saline, both for a total of 42 days. Both femoral head specimens were obtained for computed-assisted morphometry. For each rat, the right operated head was compared with the left, and the alendronate treated group was compared with the control group.

**Results:**

No differences were found in shape factor and femoral head height/length ratios in the alendronate treated femoral heads. Among the nontreated control group, shape-factor differences were found between the operated and the nonoperated femoral heads.

**Conclusion:**

Alendronate treatment prevented the distortion and destruction of the femoral head. Osteoclast inhibition might prolong the bone creeping substitution process and could enable secondary bone maturity and mineralization that increases bone strength. Alendronate preserved the femoral head architecture, which might reduce morbidity and disability due to femoral head collapse.

## INTRODUCTION

Surgical osteonecrosis of the rat femoral head is induced by detaching the ligamentum teres and stripping the femoral neck periosteum.^1^ Hematopoietic necrosis of the bone marrow is seen from the second day, and subchondral and epiphyseal bone necrosis are found on the fifth postoperative day.^1^,^2^ Inflamed reaction around the joint, especially from the joint capsule, promotes at first invasion of mesenchymal cells into the femoral head, which creates a healing process during the second week and is replaced by well vascularized fibrous tissue. This process involves macrophages and osteoclast activity, which removes the necrotic bone that is replaced by fibrous tissue and eventually by osteoblast deposit and intramembranous bone. During the remodeling process, the spherical shape of the femoral head epiphysis and physis is distorted and becomes flattened.^1^,^3^,^4^ The rapid repair process of the femoral necrosis by the newly formed bone brings early femoral head architectural distortion. These findings concerning femoral head shape changes are due to the biomechanically weaker newly deposited bone compared with the mature bone.^5^,^6^ The femoral head flattening and distortion brings osteoarthritic changes affecting the hip joint, which are some of the leading causes of total hip replacement in the young population.^6^

It is known that small changes to the structure of the R^2^ side chain of nitrogen-containing bisphosphonates can dramatically affect their potency for inhibiting bone resorption *in vitro* and *in vivo*, although the reason for these differences in antiresorptive potency have not been explained at the level of a pharmacological target. Several nitrogen-containing bisphosphonates were found to inhibit osteoclast mediated bone resorption *in vitro* by inhibiting farnesyl diphosphate synthase, thereby preventing protein prenylation in osteoclasts.^7^ Osteoblasts continue to function by enhancing bone growth, resulting in increased bone mass or density, and osteoclast suppression, which also is intended to enhance overall bone quality.^7^ Alendronate is a nitrogen-containing biphosphonate that retains the osteoclasts and, as a result, inhibits their activity, resulting in inhibition of the remodeling and healing processes of the necrotic femoral head bone.^8^–^11^

The purpose of this study was to evaluate and to determine objectively the influence of alendronate treatment on the rat femoral head shape after six weeks of daily treatment, when compared with controls.

## MATERIALS AND METHODS

**Figure 1:**
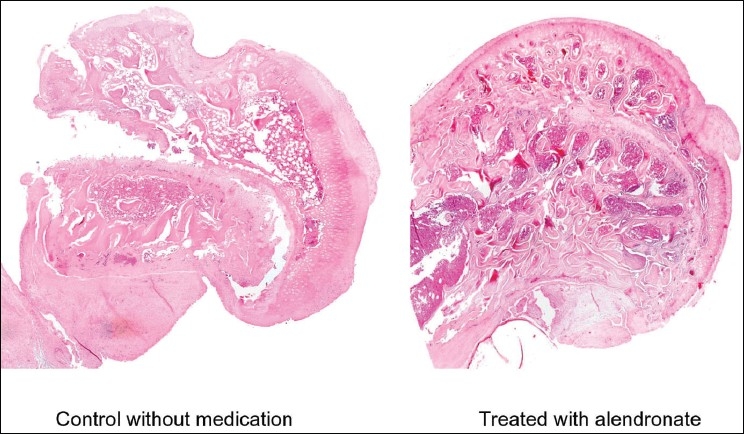
Femoral heads coronal section 42 days after surgical AVN at the teres ligament origin after H&E staining. The right is without Alendronate treatment, control, showing distortion of the spherical head shape. On the top, the weight bearing zone, the articular cartilage looses its height and is associated with arthritic changes. The epiphysis height is changed along the head and is smaller on the weight bearing zone. The left is the femoral head with Alendronate treatment. The spherical head shape is preserved and looks like normal. The cartilage height is equal all along the head and is smooth. The epiphysial height is preserved and similar along the head in the weight bearing zone (above the teres ligament), and in the non-weight bearing zone area (below the teres ligament).

Approval of local institutional review board was obtained prior to the start of the study. The blood circulation of the right femoral head of 20 female Sprague-Dawley rats, weighing 400–450 g, was interrupted. They were divided into two groups. One group was treated by daily subcutaneous injections of alendronate, and the control group was injected with saline. The treated group consisted of 12 animals, and the control group had eight animals. The animals were housed in spacious cages to allow ample ambulation and were operated after a minimum of a week for acclimation to the cage. They had free access to water and regular laboratory chow at all times. The rats were anesthetized with an intramuscular injection of ketamine (120 mg/kg) and xylazine (17 mg/kg). They were placed on a heated operating table to prevent hypothermia. After shaving of the skin, antisepsis, draping, and proximally slitting of the cutis by a longitudinal incision over the greater trochanter, the gluteus maximus muscle was split in the direction of its bundles and the anterior two-thirds of the gluteus medius muscle were detached from the bone. The anterolateral insertion of the articular capsule was transected along the trochanteric ridge, the femoral head was dislocated, and the ligamentum teres was cut. With a number 11 blade, the periosteum at the base of the neck of the femoral head was incised together with the reflected capsular fibers by circumferentially sweeping the edge of the knife twice, at a 1- mm interval, around the bone. The femoral head was relocated. The articular capsule and the gluteal muscles were sutured with vicryl 3-0 stitches. The skin was closed with nylon 2-0 stitches.^1^ A mother alendronate solution was prepared by diluting tablets of 70 mg alendronate sodium (Fosalan) (MSD Inc., Harlem, Holland) at a concentration of 500 *µ*g/mL^3^ of physiological saline and mixed in an electrical stirrer for 90 min. Postoperatively, the 12 rats in the treated group received daily subcutaneous injections of 200 *µ*g of alendronate per 1 kg of body weight in the neck region to which a saline solution was added up to a total volume of 0.5 mL. The eight control rats were treated with 0.5 mL saline instead of the alendronate solution. All rats were killed by CO_2_ inhalation on the 42^nd^ postoperative day, both femoral heads were harvested and the soft tissue was excised. The specimens were fixed in formalin for a week. Following decalcification in EDTA for two weeks, the femoral heads were halved at the residue of the ligamentum teres into anterior and posterior parts. Paraffin blocks of 4 *µ*m were cut, and the sections were stained with hematoxylin and eosin [Figure 1]. All the histologic specimans were numbered randomly.

Histomorphometric analysis of the sections was performed using a computerized image analysis system. The system consisted of a trinocular Olympus CH-40 bright-field microscope (Olympus Corporation, Tokyo, Japan) fitted with a Zeiss Plan 1× objective. This ultra-low objective allows the structures in the preparation to be examined and measured on a single image. The image passes through a 2.5× projection lens to a WAT202D digital video camera and the image is seen on a 19” video monitor using a software from Soft Imaging System, Munster, Germany (analySIS® version 3.0). The system is very accurately calibrated for the specific objective and images and all the data recorded were in micrometers.

The field of interest was chosen as the femoral head portion overlying an imaginary straight line at the insertion of the joint capsule to the cartilage-bone junction. Feret diameters were gauged at 10- degree intervals. The maximum and minimum feret diameters expressing the longest and the height of the femoral head were measured. These values were used to compare the height/length (H/L) ratio of the femoral heads. The shape-factor (SF) of the femoral heads was determined to assess the femoral head profile. The SF was defined as 4πA/P^2^, where A is the femoral head area and P is the perimeter. Round objects have SF values close to 1 while linear objects have 0 values.

To evaluate the specimens objectively and to prevent biased results, all the slides were numbered randomly and mixed. The examiner who received the slides knew that they were mixed. After completing the measurements, the slide results were uncovered. For each rat, the right femoral head was compared to the left and the alendronate-treated group was compared to the control group.

Statistical evaluation was done by a statistician. Mean, standard deviation (SD) and ranges were calculated. The Signed Ranks Wilcoxon test was used to compare between the right and the left femoral head of each animal. Mann-Whitney test was used to compare between the treated and the control groups. *P* values up to 0.05 were considered significant.

## RESULTS

The rat femoral heads were prepared and evaluated by histomorphometry using computer assistance as described earlier. We evaluated the femoral head shape using the shape-factor (SF) and the head/length ratio (H/L). The right operated head was compared with the nonoperated head in all the animals, and the alendronate-treated group was compared with the control group.

No SF differences (*P* = 0.31) were found when comparing the right and left femoral heads in the alendronate-treated group. On the other hand, SF differences (*P* = 0.01) were found when comparing the right operated and the left normal femoral heads in the control group.

When comparing the right and left femoral heads using the Wilcoxon signed ranks test, there were no differences in the treated group (*P* = 0.95).

Significant H/L ratio differences (*P* = 0.01) were found between the treated and the control groups. In the SF, differences were almost significant, with a *P* value of 0.06. No differences were found between the left femoral heads.

## DISCUSSION

The purpose of this study was to evaluate and to objectively determine the influence of alendronate treatment on the rat femoral head shape after six weeks of daily treatment compared with controls. Osteonecrosis and death of the bone marrow after an ischemic event initiates a reparative process. A lesion that is larger than 15% of the femoral head can be assumed to have difficulties in healing and might collapse.^12^ Collapse of the head occurs when the necrotic bone underlies the weight-bearing region, and when subchondral bone is flattened it is predictive of pending collapse.^13^,^14^

The femoral head sphericity, as represented by the SF [Table 1] did not differ (*P* = 0.31) when comparing the operated right femoral head with the left unoperated femoral head in the alendronate-treated group. In the control group, we found significant SF differences (*P* = 0.01) when comparing the right operated femoral head with the left normal side. These finding were very similar when we compared the H/L ratios [Table 2], and there were no differences (*P* = 0.95) between the operated and nonoperated femoral heads in the alendronate-treated group. In the control group, major femoral head deformities will probably bring joint destruction and osteoarthritis of the hip joint [Figure 1]. Among the alendronate-treated group, there were no differences between the right operated and the left nonoperated femoral heads. By treating the animal with alendronate, we preserved the femoral sphericity.

**Table 1:** Femoral heads shape-factor of the two groups

		Treated group *n* = 12	Control group *n* = 8
Right femoral head	Mean ± SD	0.61 ± 0.05	0.55 ± 0.06
	Range	0.49–0.67	0.42–0.62
Left femoral head	Mean ± SD	0.64 ± 0.02	0.65 ± 0.04
	Range	0.59–0.66	0.57–0.72
*P* value (right/left)		0.31	0.012

shape-factor = 4πA/P^2^ where A is the femoral head area and P is the perimeter; *P* value was calculated by comparing the right and left femoral heads using Wilcoxon signed rank test.

**Table 2:** Femoral heads height/length ratios of the two groups

		Treated group *n* = 12	Control group *n* = 8
Right femoral head	Mean ± SD	0.50 ± 0.06	0.448 ± 0.09
	Range	0.37–0.65	0.31–0.62
Left femoral head	Mean ± SD	0.51 ± 0.04	0.520 ± 0.07
	Range	0.42–0.57	0.43–0.66
*P* value (right/left)		0.95	0.16

*P* value was calculated by comparing the right and left femoral heads using Wilcoxon signed rank test.

When comparing the groups, significantly higher H/L ratios were found in the alendronate-treated group. The SF changes between the groups were almost significant (*P* = 0.06); by enlarging the experimental group, it would be expected that the changes would be significant. Nishii *et al.* compared the influence of alendronate treatment in 22 patients with osteonecrosis of the femoral head for a year. Twenty hips of 14 patients were treated on a daily basis with alendronate, compared to 13 hips of eight patients the control group. All patients had measurements of biochemical markers of bone turnover at entry into the study, and the patients in the alendronate group repeated the measurements at 3, 6, and 12 months. The alendronate group showed a greater decrease of biochemical markers of bone resorption, a lower frequency of collapse of the femoral head, and reported less hip pain than the control group.^15^ Lai *et al.* reported about 40 patients with femoral head AVN with a necrotic area greater than 30%. Half the patients were treated with alendronate once a week for 25 weeks and compared with untreated control patients. After a minimum of two years of follow-up, two of 29 femoral heads in the alendronate group collapsed, one of which underwent total hip replacement, whereas 19 of 25 femoral heads in the control group collapsed, 16 of which needed total hip replacement.^16^ Ramachandran *et al.* evaluated intravenous bisphosphonate therapy for femoral head osteonecrosis following trauma in 17 adolescents. Treatment duration was up to 39 months, and the patients were followed radiographically and clinically for a minimum of two years. All the 17 patients had a good or excellent outcome.^17^ Increasing the revascularization with more advanced bone resorption and regeneration as reported in a previous study brought increased bone collapse.^18^

We assume that, by slowing down bone resorption using osteoclasts inhibitor, such as alendronate, neovascularization and new bone regeneration will slow down. The necrotic bone structure and strength is preserved, and it might be able to “carry” the loads passing through the femoral head. Slowing down of the necrotic bone creeping substitution process will allow the newly formed bone to become stronger to an amount that will carry the load passing along the femoral head. This study might bring a partial histological explanation supporting the literature concerning alendronate treatment for femoral head osteonecrosis.^9^–^11^,^15^–^18^

On the other hand, bone resorption inhibitor therapy, including alendronate, is associated with mandibular and maxillar osteonecrosis and the mechanism of this adverse effect is poorly understood.^19^,^20^

In this study, we did not estimate the percentage of the necrotic bone or the newly formed bone. The femoral head strength was not evaluated, nor were other effects, if any, on different bones in the animal skeleton.

## CONCLUSIONS

We found that alendronate treatment has the potential to prevent critical mechanical femoral head weakening by slowing down the necrotic bone healing process. This slowing down process will probably bring a longer period of healing and bone recovery. It will prevent femoral head deformities, collapse, and most probably preserve femoral head sphericity, and will keep the hip joint pain free.

## References

[1] Norman D, Reis D, Zinman C, Misselevich I, Boss JH (1998). Vascular deprivation-induced necrosis of the femoral head of the rat: An experimental model of avascular necrosis in the skeletally immature individual or Legg-Perthes disease. Int J Exp Pathol.

[2] Boss JH, Misselevich I (2001). Review: Experimental avascular osteonecrosis. Curr Orthop.

[3] Levin D, Norman D, Zinman C, Misselevich I, Reis NR, Boss JH (1999). Osteoarthritis-like disorder in rats with vascular deprivation-induced necrosis of the femoral head. Pathol Res Prac.

[4] Peskin B, Shupak A, Misselevich I, Zinman C, Levin D, Jacob Z (2001). Transphyseal osseous bridges in experimental osteonecrosis of the femoral head of the rat: Histologic study of the bony bridges connecting the epiphyseal with the metaphyseal bony trabeculae through gaps in the physeal cartilage. J Pediatr Orthop Br.

[5] Boss JH, Misselevich I, Bejar J, Norman D, Zinman C, Reis DN (2004). Experimentally gained insight-based proposal apropos the treatment of osteonecrosis of the femoral head. Med Hypotheses.

[6] Peled E, Boss JH, Norman D, Bejar J, Ben-Noon H, Zinman C (2006). Effects of alendronate medication on the fate of the necrotic femoral head of rats with or without core decompression. J Orthop Traumatol.

[7] Dunford JE, Thompson K, Coxon FP, Luckman SP, Hahn FM, Poulter CD (2001). Structure-activity relationships for inhibition of farnesyl diphosphate synthase in vitro and inhibition of bone resorption in vivo by nitrogen-containing bisphosphonates. J Pharmacol Exp Ther.

[8] Reszka AA, Rodan GA (2004). Nitrogen-containing bisphosphonate mechanism of action. Mini Rev Med Chem.

[9] Agarwala S, Sule A, Pai BU, Joshi VR (2001). Study of alendronate in avascular necrosis of bone. J Assoc Physicians India.

[10] Agarwala S, Sule A, Pai BU, Joshi VR (2002). Alendronate in the treatment of avascular necrosis of the hip. Rheumatology.

[11] Agarwala S, Jain D, Joshi VR, Sule A (2005). Efficacy of alendronate, a bisphosphonate, in the treatment of AVN of the hip: A prospective open-label study. Rheumatology.

[12] Mont MA, Jones LC, Einhorn TA, Hungerford DS, Reddi AH (1998). Osteonecrosis of the femoral head: Potential treatment with growth and differentiations. Clin Orthop Relat Res.

[13] Sabo E, Peskin B, Misselevich I, Zinman C, Levin D, Norman D (2001). Computer-assisted image analysis of the rat postosteonecrotic remodeled femoral head. Exp Mol Pathol.

[14] Ohzono K, Saito M, Takaoka K, Ono K, Saito S, Nishina T (1991). Natural history of nontraumatic avascular necrosis of the femoral head. J Bone Joint Surg Br.

[15] Nishii T, Sugano N, Miki H, Hashimoto J, Yoshikawa H (2006). Does alendronate prevent collapse in osteonecrosis of the femoral head?. Clin Orthop Relat Res.

[16] Lai KA, Shen WJ, Yang CY, Shao CJ, Hsu JT, Lin RM (2005). The use of alendronate to prevent early collapse of the femoral head in patients with nontraumatic osteonecrosis: A randomized clinical study. J Bone Joint Surg Am.

[17] Ramachandran M, Ward K, Brown RR, Munns CF, Cowell CT, Little DG (2007). Intravenous bisphosphonate therapy for traumatic osteonecrosis of the femoral head in adolescents. J Bone Joint Surg Am.

[18] Levin D, Norman D, Zinman C, Rubinstein L, Sabo E, Misselevich I (1999). Treatment of experimental avascular necrosis of the femoral head with hyperbaric oxygen in rats: Histological evaluation of the femoral heads during the early phase of the reparative process. Exp Mol Pathol.

[19] Benhamou CL (2007). Effects of osteoporosis medications on bone quality. Joint Bone Spine.

[20] Levin L, Laviv A, Schwartz-Arad D (2007). Denture-related osteonecrosis of the maxilla associated with oral bisphosphonate treatment. J Am Dent Assoc.

